# Assessing the risk for suicide in schizophrenia according to migration, ethnicity and geographical ancestry

**DOI:** 10.1186/s12888-016-1180-3

**Published:** 2017-02-09

**Authors:** Nuwan C. Hettige, Ali Bani-Fatemi, James L. Kennedy, Vincenzo De Luca

**Affiliations:** 1 Institute of Medical Science, University of Toronto, Toronto, M5S 1A8 Canada; 2 Centre for Addiction and Mental Health, 250 College Street, M5T 1R8 Toronto, Canada; 3 Department of Psychiatry, University of Toronto, Canada, 250 College Street, Toronto, M5T 1R8 Canada

**Keywords:** Schizophrenia, Suicide, Migration, Ethnicity, Ancestry

## Abstract

**Background:**

Suicide is a leading cause of mortality among those afflicted by schizophrenia. Previous studies demonstrated that the stressors associated with immigration may lead to an onset of schizophrenia and suicide separately in susceptible individuals. However, no studies have shown whether immigration may lead to suicidal behaviour for individuals with schizophrenia. Our study proposes that an individual’s geographical ancestry, ethnicity or migration status may be predictive of suicide risk in schizophrenia.

**Methods:**

In a sample of 276 participants with schizophrenia spectrum disorders, we conducted cross-sectional assessments to collect clinical information. Self-identified ethnicity and suicide history were collected through self-report questionnaires and interview-based scales. Ancestry was identified using 292 genetic markers from HapMap. Migrants were classified as those who immigrated to Canada during their lifetime. Using a regression analysis, we tested whether a history of migration, ethnicity or geographical ancestry were predictive of a history of suicide attempts.

**Results:**

Our analysis failed to demonstrate a significant relationship between suicide history and migration, ethnicity or ancestry. However, ethnicity appears to be significantly associated with the number of psychiatric hospitalizations in our sample.

**Conclusion:**

Ethnicity and migration history are not predictive of previous suicide attempts. Ethnicity may be an important demographic factor affecting access to mental health resources and frequency of hospitalizations.

## Background

Suicide is a complex behaviour and a major public health concern, ranking among the leading causes of death in Canada [1]. Despite being an avoidable outcome, suicide continues to contribute to the mortality of many individuals around the country. Sadly, due to the stigma and prejudice surrounding suicidal behaviour, many individuals fail to reach out or seek help when faced with these difficulties. Suicidal behaviour is generally defined along a continuum that includes suicidal ideation and suicide attempt, with the extreme end of the continuum being suicidal completion [2]. Unfortunately, due to the complex nature of suicide, it is difficult for clinicians to identify individuals at the highest risk.

Many individuals that attempt suicide often experience severe despair frequently attributed to a psychiatric disorder. In fact, schizophrenia (SCZ), a common psychiatric disorder, has been shown to increase an individual’s risk for suicide [3]. In Canada, we are arguably witnessing an increase in the rate of SCZ and higher prevalence and incidence rates compared to some international populations [4]. SCZ may potentially arise due to a combination of environmental and genetic factors. Generally, SCZ onset is around late adolescence and early adulthood, producing a functional decline and then chronic impairment [5]. SCZ, which is a major cause of disability, has severe consequences on patients and their relatives such as economic, health, and personal issues, especially in those with no sufficient treatment.

Individuals with SCZ generally experience a range of symptoms, such as delusions, sensory hallucinations, flat or blunted emotions, and disorganized thoughts and speech [6–8]. Accordingly, SCZ is associated with a significantly increased risk for self-injurious and suicidal behaviours [9]. In particular, suicide attempts and suicide completion are one of the largest contributors to the increased morbidity and mortality rates in SCZ, respectively [10]. Recent epidemiological studies have identified that the current suicide risk for patients with SCZ is approximately 4.9% [11] or 0.24 per 100 person years [12]. It has also been proposed that approximately between 20 and 40% of patients diagnosed with SCZ will attempt suicide during the duration of their illness [13]. Sadly, attempting to identify individuals with SCZ at the highest risk for suicide remains a major obstacle. Some studies have proposed that an increased incidence of SCZ is associated with some ethnic minorities and migration status [14–16].

It has been previously reported that immigrant populations has an increased risk for developing SCZ, suggesting that social factors play a vital role in the pathology of SCZ [17, 18]. Notably, belonging to a visible minority group also leads to a higher risk for developing SCZ. On the other hand, being part of a high ethnic density of a minority group in a specific neighbourhood can be a protective factor [19–21]. Therefore, it has been proposed that rather than the stressors of migration, it is “the exception to the norm” that contributes to the increased risk for SCZ [22, 23]. Interestingly, a meta-analysis by Cantor-Graee and Selten [24] found that the mean weighted relative risks of SCZ were 2.7 and 4.5 times higher for first and second generation immigrants, respectively. Cantor-Graee [18] proposed that social defeat may be a plausible mechanism for explaining these elevated risks, whereby social defeat is defined as an experience of being in a subordinate or outsider position.

The rates of psychosis and suicide among immigrants vary markedly depending on the country where the immigrant population resides [25]. The experiences that come along with immigration are highly heterogeneous and not all immigrants may face similar situations before or after migration [26]. Settling in a new country can be an extremely stressful situation for the individual, potentially causing severe psychological distress and trigger suicidal behaviours. Many individuals face drastic changes in social roles and social status, and are potentially exposed to social marginalization, prejudice, and discrimination by the host population [27, 28]. In comparison to the studies conducted so far on suicide and immigration, regrettably only a few studies have focused on attempted suicide among immigrants.

Previous research suggests that the immigrant population is at a higher risk of developing SCZ and suicidal behaviours [29, 30]. Identifying a risk for suicide attempt that is due to factors associated with immigration and ethnicity can help clinicians pinpoint prevention efforts. This is particularly important and relevant for ethnically diverse countries such as Canada, where studies have shown that immigrant and ethnically diverse populations show differences in suicide completion and suicide attempts [4]. Therefore, the aims of this study are to predict whether individuals with SCZ are more likely to have attempted suicide if they have immigrated to Canada during their lifetime and whether differences in self-reported ethnicity or genetically-determined geographical ancestry are predictive of a history of suicide attempts. Given the current literature on the topic, it is hypothesized that individuals with SCZ who have immigrated to Canada and are of an ethnically different background from the visible majority in Canada (European Caucasian) are more likely to have a history of attempted suicide.

## Methods

### Subjects and assessments

We originally recruited 336 participants between the ages of 18 to 75 from the Centre for Addiction and Mental Health (CAMH) in Toronto. All patients met the criteria for schizophrenia spectrum disorders based on the structured clinical interview for DSM-IV (SCID-I/P) [31] (American Psychiatric Association, 2013). Inclusion criteria included a diagnosis of SCZ or schizoaffective disorder while our exclusion criteria included evidence of intellectual disability and the presence of neurodegenerative disorders. In addition, those who have experienced brain injury trauma with a loss of consciousness and a history of major substance abuse prior to the onset of illness were excluded to ensure that the onset of the participant’s symptoms was not directly attributed to the intake of drugs or physical problems. Written informed consent was obtained for participation in the study as well as for the release of participants’ medical history in order to verify their oral accounts or obtain missing information. This study was approved by the CAMH Research Ethics Board.

Assessments were conducted cross-sectionally using a structured interview and self-report questionnaires. The interview incorporated the Structured Clinical Interviews for DSM-IV (SCID-IV) in order to diagnose participants, as well as to assess for additional psychiatric symptoms and comorbid diagnoses. In situations where a diagnosis could not be reliably defined, the individual was excluded from the analysis. Information regarding the participants’ ethnicity was also collected through self-report and interview questions. Ethnicity was defined by identifying the ethnic background of the participants’ four grandparents and through written self-report. Individuals were classified as either European Caucasian or Other. Cases in which there was ambiguity or discrepancy in the individual’s ethnicity lead to them being excluded.

During the clinical assessment, participants’ suicidal history was assessed using the Beck Suicide Ideation Scale (BSS) [32] and the Columbia Suicide Severity Rating Scale (C-SSRS) [33]. According to the patients’ response on the BSS and C-SSRS, they were classified as suicide attempters (those who have attempted suicide at least once in their lifetime) and non-attempters (those who never attempted suicide in their lifetime). Individuals with an ambiguous or ill-defined suicide attempt history were excluded.

Our original sample size consisted of 336 individuals diagnosed with either SCZ or schizoaffective disorder; though our final analysis included 276 participants after filtering criteria were applied. Participants not included in our analysis were those who had inconsistent, incomplete or inaccurate reports of previous suicide attempts and an ambiguous or unclear ethnic background.

### Clinical analysis on suicide attempt history

All data analyses were performed using R (v.3.1.3) [34]. Participants were assessed according to two groups: Suicide Attempters and Non-Attempters. The presence of at least one suicide attempt lifetime versus patients who never attempted suicide was used as the main grouping variable. Attempters and non-attempters were compared to test for significant differences regarding age, sex, ethnicity and migrant status. A summary of the clinical variables can be found in Table 1. We used a logistic regression model to determine whether differences in migrant status (immigrants versus native-born individuals) and ethnicity (European Caucasian versus Others) were predictive of a history of previous suicidal attempts. Our tests of significance were done with a confidence interval of 95% with an alpha level of 0.05.

**Table 1:** Participant Clinical Demographics

Total (*N* = 276)	Suicide Attempters (*N* = 126)	Non-Attempters (*N* = 150)
Age (X, SD)	39.74 (11.42)	40.56 (12.42)
Age of Onset (X, SD)	22.04 (6.71)	22.52 (7.26)
Sex (Male/Female)	91/35	96/54
Migration Status (Migrant/Native)	29/97	35/115
Hospitalizations (X, SD)	5.98 (6.95)	4.29 (5.23)
Comorbid Alcohol Use (%)	37%	28%
Current smokers	54%	51%
Comorbid Drug Use (%)	31%	29%
Marijuana Use (%)	25%	25%
Duration of Illness (years) (X, SD)	17.73 (11.00)	18.42 (11.70)
Self-Reported Ethnicity (European Caucasian/Other)	101/25	112/38

### Genetic analysis of geographical ancestry

Our sample consisted of 155 subjects genotyped using a customized Illumina Bead Chip and 121 subjects genotyped using the Illumina Omni-2.5 array. Genotype imputation was conducted for the 121 subjects using IMPUTE2 (v.2.3.1) and 1000 Genomes Phase 3 reference data to obtain a selection of 292 single nucleotide polymorphism (SNP) ancestry-informative markers from an original panel of 384 markers represented in the HapMap Phase II project [35]. Three reference populations were used from the HapMap project: European Caucasians (North/Western Europeans from Utah [CEU]), East Asians (Han Chinese and Japanese individuals [CHB + JPT]), and Africans from Nigeria (Yoruba from Nigeria [YRI]).

**Fig. 1:**
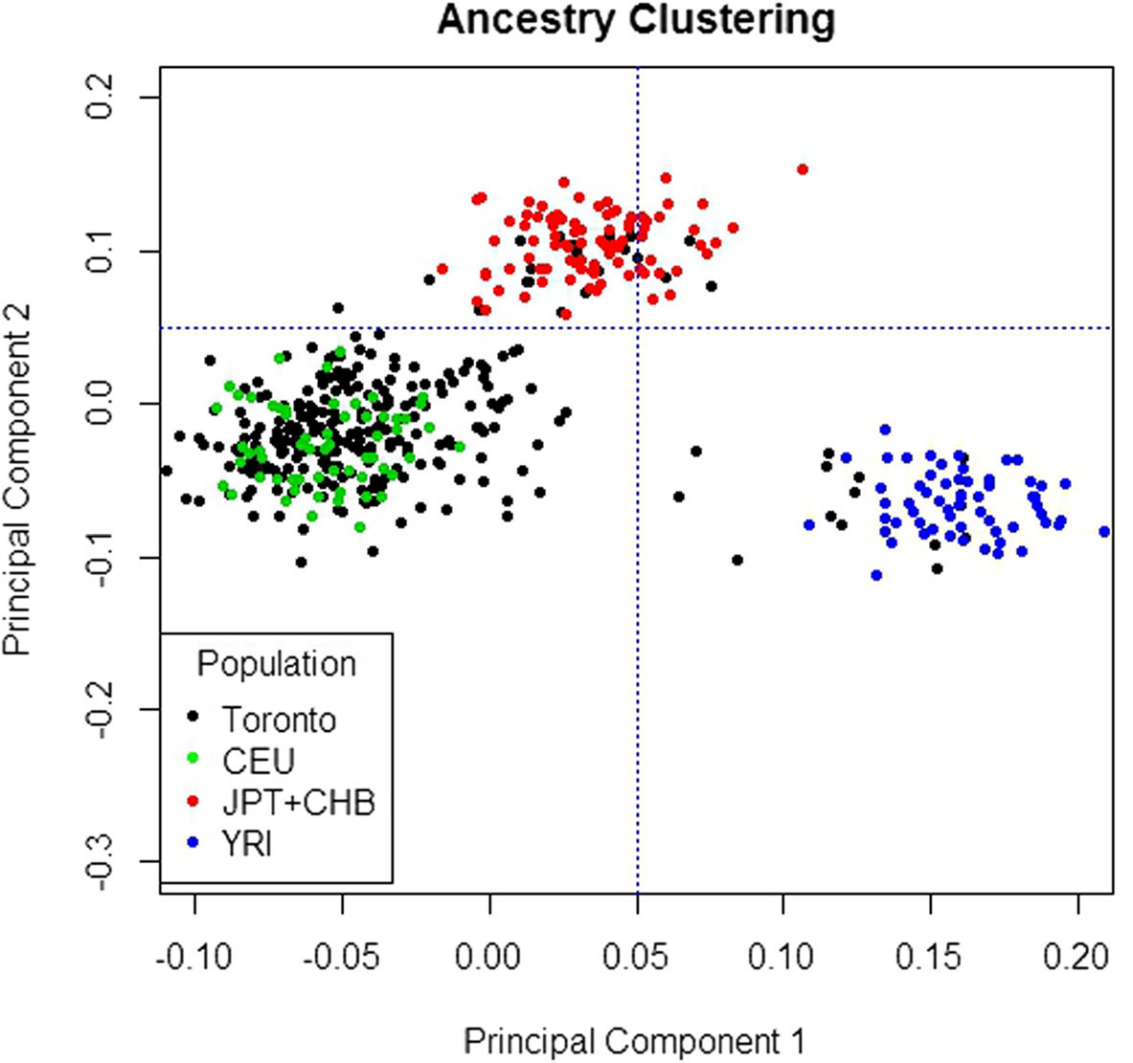
Multidimensional scale clustering according to the geographical ancestry using 292 SNP markers from the HapMap Phase II project

We determined ancestry with the Multidimensional Scaling (MDS) procedure using PLINK v1.07. The first two components were used to identify population stratification. We plotted the two components for all participants in our study sample relative to the individuals from the three major ethnic clusters from HapMap II as seen in Fig. 1. Ancestry was determined using the 292 SNP markers. Self-reported ethnicity in all our participants was confirmed using the MDS plot. Individuals who self-reported as European Caucasian and Other ethnicity were visually distinguishable. We excluded individuals with discordant self-reported ethnicity and geographical ancestry, in which self-reported ethnicity did not match geographical ancestry according to the MDS. Parameter were drawn at x = 0.05 to distinguish European Caucasians from those of African ancestry, and y = 0.05 to separate European Caucasians from those of East Asian ancestry. We then performed a second regression analysis for suicide attempt history and ethnicity with a more precise ethnic background.

**Fig. 2:**
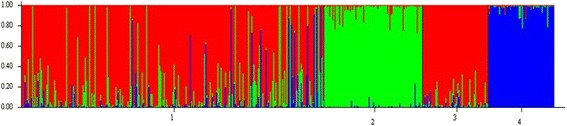
STRUCTURE analysis of self-reported ethnicity of study participants and reference population from HapMap.1 = Study participants (*n* = 276); 2 (*green*) = Japanese and Han Chinese Asians (JPT + CHB) reference population (*n* = 90); 3 (*red*) = North/western Europeans from Utah (CEU) reference population (*n* = 60); 4 (*blue*) = Yorubans from Nigeria (YRI) reference population (*n* = 60)

To test whether geographical ancestry was predictive of suicide attempt history we used STRUCTURE (v.2.3.4) [36] to identify population substructure and estimate the geographic ancestry of the study participants (Fig. 2). The three HapMap Phase II populations were also included. The output from STRUCTURE provides the relative ethnic proportion for each individual, namely the percentage that each individual was of CEU, CHB + JPT, and/or YRI ancestry. As our analysis considered ethnicity as European Caucasian or Other, we considered the proportion that each individual was of European ancestry in our regression model to test whether it was predictive of a history of previous suicide attempts.

## Results

Our sample of 276 participants consisted of 186 males and 90 females. There were 126 individuals with a history of attempted suicide, 97 of which were native-born while 29 were immigrants to Canada. Furthermore, there were 150 individuals who have never attempted suicide, including 115 native-born and 35 immigrants. As we chose to categorize the ethnicity of our participants as either European Caucasian or Other, our sample consisted of 213 self-reported European Caucasians and 63 individuals of varied ethnic backgrounds. After correcting ethnicity for geographical ancestry according to our MDS plot, our sample size included 247 individuals.

In the analysis of the clinical variables, our logistic regression analysis demonstrated non-significant results in predicting suicide attempt history for all observed outcomes: age of symptom onset (OR = 0.99; 95% CI: 0.96 - 1.02; *p* = 0.570), gender (OR = 1.46; 95% CI: 0.879 – 2.46; *p* = 0.146), age (OR = 0.99; 95% CI: 0.97 – 1.01; *p* = 0.437). Furthermore, we found that migration status did not significantly predict the number of hospitalizations (OR = 0.97; 95% CI: 0.91 – 1.02; *p* = 0.295). On the other hand, ethnicity was significantly predicted by the number of hospitalizations (OR = 1.11; 95% CI: 1.03 – 1.21; *p* = 0.011) such that being of a European Caucasian ethnic background predicted a higher number of hospitalizations.

In our analysis, we considered whether migration status (being native-born to Canada or an immigrant) was predictive of a history of suicide attempt. We found that there was no significant association between migration status and suicide (OR = 0.98; 95% CI: 0.56–1.72; *p* = 0.950). Furthermore, when we considered whether ethnicity (European Caucasian versus Other ethnic backgrounds) was predictive of suicide attempt history, we found no significant association (OR = 1.37; 95% CI: 0.78 – 2.45; *p* = 0.280). When we performed a second regression analysis considering self-reported ethnicity, in which individuals with discordant ethnicities and geographical ancestries were excluded according to our MDS plot, we similarly found no significant relationship (OR = 1.89; 95% CI: 0.89 – 4.20; *p* = 0.105) between ethnicity and suicide attempt history. Additionally, we continued to see that the number of hospitalizations was significantly predicted by ethnicity according to self-report and confirmed by geographical ancestry (OR = 1.05; 95% CI: 1.01 – 1.10; *p* = 0.040)

Finally, we wanted to test whether geographical ancestry was able to reliably predict suicide attempt history. Figure 2 illustrates the STRUCTURE analysis of study participants (Study participants, *n* = 276; reference population of CEU, *n* = 60; reference population of JPT + CHB, *n* = 90; and reference population of YRI, *n* = 60). Ethnicity according to our STRUCTURE analysis was not associated with a history of previous suicide attempts in our SCZ population (OR = 0.18, 95% CI: − 0.025 – 0.377, *p* = 0.088).

## Discussion

In the current study, we aimed to determine whether migration status, ethnicity and geographical ancestry significantly predict a history of suicide attempts. From our analysis, we found no significant association between self-reported ethnicity (European Caucasian versus Other ethnicity) or migration status (migrant versus native-born) with previous suicide attempt history. After correcting self-reported ethnicity according to our MDS plot, there continued to be no significant association with suicide attempt history. Interestingly, however, we found that self-reported ethnicity significantly predicted the number of hospitalizations, such that those of a self-reported European Caucasian ethnicity had a higher number of hospitalization visits compared to non-self-identified European Caucasians. Finally, we found that ancestry according to our STRUCTURE analysis was also not predictive of past suicide attempts.

Here we report that self-reported European Caucasian ethnicity is predictive of higher psychiatric hospitalizations. It is possible that the lower usage rates of mental health services by non-Caucasian individuals is a result of cultural and language barriers [37]. This may be especially true for immigrant populations that face greater barriers to healthcare access; however, we found no significant relationship in this regard. In contrast, in a systematic review by Bhui et al. [38], it was reported that overall individuals of African ancestry had the highest rates of inpatient admission. On the other hand, Cole et al. [39] demonstrated similar results to our own in which Caucasians had higher rates of hospitalizations. A controversial issue, there have been differences in psychiatric admission rates in the United Kingdom in the minority population compared to the European Caucasian majority [40, 41]. In culturally diverse populations it is important to address the issue of ethnicity and its role in mediating the efficacy of mental health services.

The finding that psychiatric hospitalizations significantly differ according to ethnicity is important for suicide studies as hospital admissions are an important risk factor for suicide in SCZ. The number of hospitalizations is typically representative of the clinical functioning of the patient. Notably, it has been found that the risk for a suicide attempt is highest following discharge [42]. Furthermore, approximately one-third of hospitalized patients attempt suicide during admission or following one week after discharge. The risk for suicide following a discharge has also been shown to last as long as one year [43].

It is possible that ethnicity has some influence on the direction, quality and frequency of mental health services. Ethnicity typically dictates a unique social group identified by language, religion and birth place [44]. As a result, there could be barriers between different ethnic groups that mediate decisions on seeking help for psychiatric concerns and the resources that are available to them [45, 46]. Additionally, interactions with health care providers may be influenced by perceived or actual differences in ethnic groups [47]. Many studies have previously reported differences between ethnic groups in relation to access to mental health services; however, it is important to be cognizant of other factors such as socioeconomic status, discrimination, and language barriers that also influence health care access [48, 49].

When interpreting studies that aim to identify a relationship with ethnicity such as the current study, it is important to be aware of how ethnicity is defined. Here, we initially identified ethnicity according to participants’ self-report when performing our analyses. In this case, self-reported ethnicity may more likely be dependent upon culture. However, we also took further steps to identify ethnicity by incorporating geographical ancestry as a biological indicator of ethnicity by using genetic markers. By looking at whether self-reported ethnicity matched that of biological geographical ancestry in our sample we found 29 individuals who self-reported as being non-European Caucasian but clustered in the European ancestry sample. Upon closer inspection, majority of these individuals were of East Indian ancestry. Therefore, it is evident that the number of genetic markers used in this analysis was not sufficient to clearly separate populations with more closely shared ancestry. While ethnicity according to both self-report and biological ancestry were not significantly predictive of suicide attempt history, this procedure offers a novel and more robust measure of defining ethnicity for future studies of this nature. Future studies, should however, incorporate a large number of ancestry-informative markers to reliably cluster individuals according to geographical ancestry.

Lastly, our analysis failed to demonstrate predictive value for previous suicide attempt history when considering patients’ migration status. The act of immigrating has been thoroughly studied as a risk factor for the development of SCZ; however, it appears in our sample that this stressor may not further predict suicide attempts in SCZ.

Previous studies have been suggested that immigrants from countries with low suicide rates have a tendency to preserve those in their new country [50–52]. Similarly, immigrants from nations with high suicide rates show the same consistency. However, other studies have reported that suicide rates in immigrants are mainly higher compared to their native land rates [53, 54]. To add to the complexity of migration and suicide, some studies show that immigrant suicide rates are lower or converge over time with those of the host country [55]. One prevailing theory suggests that some of the immigrants who have suicidal behaviour in the new country had attitudes toward suicide in their country of birth, as effect of a genetic diathesis manifesting at times of severe distress [52].

Acculturation is a stressful process that is often accompanied with negative emotions and behaviours leading resulting in psychotic symptoms [56]. Individuals who choose to immigrate to a new country are faced with the daunting task of having to start anew and therefore may experience severe changes in their social and financial status. Social factors associated with immigration and pre-immigration can have drastic psychological implications on many individuals [40, 57]. The pre-immigration social vulnerabilities, employable skills, and identity of immigrants play a significant role on their psychological state. Following immigration, the degree of difficulty in constructing meaningful relationships and maintaining financial or social support can further reduce the psychological well-being of immigrants [57, 58].

Unfortunately, due to the nature of this study, it is difficult to disentangle the effect ethnicity and migrant status has on suicide in SCZ. As immigrants also belong to many visible minority groups, it is unclear which categorization has stronger predictive value for suicide attempts. Furthermore, aggregating immigrant groups as a singular entity can mask significant variations between different immigrant populations. The type and process of migration of course has a role, particularly for immigrants who are refugees or asylum seekers [30]. As such, comparisons of immigrant suicide rates with native populations have produced divergent results. Of course, it is important to remain cognizant of the fact that immigration and ethnicity comprise a multitude of additional factors that were not captured in this study. It is believed that religious beliefs, language and living conditions play a significant role in the perceived stress off ethnic minority and immigrant groups.

## Conclusions

In conclusion, our study demonstrates that schizophrenics who are immigrants to Canada and from a visible minority are not at a greater risk for suicide attempts. However, ethnicity has been shown to influence the number of psychiatric hospitalizations and potentially access to mental health services in our Canadian population. We also present a novel approach of implementing the MDS analysis to confirm ethnicity according to informative SNP markers. By using informative markers, we were able to accurately identify the ethnic origin of our sample and determine whether ancestry defined by biological terms is able to predict suicidal behaviour. While we failed to demonstrate any significant findings, migrant studies are uniquely informative for investigating the genetic and environmental influences on suicidal behaviour in SCZ.

## Abbreviations

BSS: Beck suicide ideation scale
CAMH: Centre for addiction and mental health
CEU: North/Western Europeans from Utah
CHB: Han Chinese individuals
CI: Confidence interval
C-SSRS: Columbia suicide severity rating scale
DSM-IV: Diagnostic and statistical manual of mental disorders – fourth version
JPT: Japanese individuals
MDS: Multidimensional scaling
OR: Odds ratio
R (v.3.1.3): R language version 3.1.3
SCID: Structured clinical interview for DSM disorders
SCZ: Schizophrenia
SD: Standard deviation
SNP: Single nucleotide polymorphism
YRI: Yoruba from Nigeria
